# Patterns and correlates of post-abortion complications in India

**DOI:** 10.1186/s12905-023-02254-x

**Published:** 2023-03-09

**Authors:** Manas Ranjan Pradhan, Daisy Saikia

**Affiliations:** 1 Department of Fertility and Social Demography, International Institute for Population Sciences, Govandi Station Road, Deonar, Mumbai, India; 2 Research Scholar, International Institute for Population Sciences (IIPS), Mumbai, India

**Keywords:** Abortion, Complications, Correlates, India

## Abstract

**Background:**

Abortion complications can range from minor and treatable to severe but rare complications that can result in morbidity or even death. There is limited evidence on the socioeconomic and demographic correlates of post-abortion complications, though abortion is associated with pregnancy and birth-related complications and contributes to maternal mortality in India. This study thus assesses the patterns and correlates of post-abortion complications in India.

**Methods:**

This study gathered data from the cross-sectional National Family Health Survey(2019–21) on women aged 15–49 who had their last pregnancy terminated by induced abortion in the five years preceding the survey (n = 5,835). Multivariate logistic regression was used to check the adjusted association of socioeconomic and demographic characteristics with abortion complications. The data were analysed using Stata with a 5% significance threshold.

**Results:**

Post-abortion complications affected 16% of the women. Women who had an abortion with a gestational age of 9–20 weeks (AOR:1.48, CI: 1.24–1.75) and those who had an abortion due to life risk/medical reasons (AOR:1.37, CI:1.13–1.65) had higher odds of abortion complications than their respective counterparts. Women in the North-Eastern (AOR:0.67, CI:0.51–0.88) and the Southern (AOR:0.60, CI:0.44, 0.81) regions were less likely to have abortion complications than those in the Northern region.

**Conclusion:**

Many Indian women suffer from post-abortion complications, with the primary causes being increased gestational age and abortions performed due to life-threatening or medical conditions. Efforts to educate women about early abortion decision-making and improve abortion care will reduce post-abortion complications.

## Background

India witnessed about 16 million abortions, 73% outside health facilities in 2015 [1]. In the country, abortions are performed for several reasons, including restricted contraception use, financial constraints, already having too many children, religious issues, abortion’s legal status, unwanted pregnancies, pregnancy as a result of rape, and risks to the mother or fetal health [2–4]. Abortion is a highly safe procedure with low risks when carried out in a safe environment and according to legal procedures [5]. However, many Indian women seek unsafe abortions [6–9]. A recent study covering nine states further reveals that 67% of the reported abortions by women are unsafe, although this statistic is likely underreported [10]. Even though abortion is legal in the country under the Medical Termination of Pregnancy (MTP) act of 1971, women frequently choose unqualified doctors and nurses to end their pregnancies [7] due to numerous individual and community-level factors, including lack of knowledge regarding the legality of abortion, limited understanding of the risks associated with unsafe abortion, a lack of safe providers and methods, poor agency and self-efficacy among women seeking abortion services, and myths, misconceptions, and social stigma associated with abortion [11–15].

Unsafe abortion contributes to morbidity and mortality [10, 16] and is considered a major public health problem in India [9, 10, 17]. Past studies have found that the frequency of unsafe abortion and its complications is high among Indian women in the reproductive age range [1, 6, 7, 9]. Complications range from those that are minor and can be easily treated to those that, while rare, are severe and can result in morbidity or even death [18]. In 2015, an estimated 5.2 million women received treatment for induced abortion complications nationally, a treatment rate of 15.7 per 1000 women aged 15–49. Approximately half of the post-abortion patients were treated for an incomplete abortion resulting from medication abortion; many of these patients may not have needed treatment to complete their abortion [8]. Potential complications related to abortions may result in pain, prolonged or abnormal bleeding, injury/perforation/laceration, sepsis, shock, irregular menstruation, incomplete abortion from a medication abortion, incomplete abortion from any other procedure, or infections of the upper genital tract that result in endometritis, oophoritis, parametritis, and salpingitis [8, 19, 20]. An ascending bacterial infection, such as chlamydia, gonorrhea, mycoplasma, and bacterial vaginosis, starts in the lower genitalia and travels up the cervix to the uterus, is frequently to responsible for infections connected to abortions [21]. The infection, if untreated, can spread to the fallopian tubes and may lead to infertility. Women who aborted are more than twice as likely to give birth to a baby early in the pregnancy (26 weeks gestation); the hazards were 71% greater at 28 weeks of gestation and 45% at 32 weeks of gestation [22].

Although abortion is connected to pregnancy and birth-related complications and is a major factor in maternal mortality, empirical evidence on socioeconomic and demographic correlates of abortion complications is limited in the country. To the best of our understanding, no past study presents the association of reason for abortion, timing of abortion, and method of abortion with abortion complications in India using large-scale representative data. This study assesses the patterns and correlates of post-abortion complications in India based on the most recent data.

## Methods

### Data

The study used data from the National Family Health Survey–5 (NFHS–5) conducted during 2019-21. The NFHS-5 is a nationally representative survey of 636 699 households that provides data for various health, nutrition, and women’s empowerment monitoring and impact evaluation indicators. The survey sample is a stratified two-stage with an overall 98% response rate. Probability proportional to size (PPS) sampling was used to select the primary sampling units: survey villages in rural areas and census enumeration blocks in urban areas. Trained research investigators used computer-assisted personal interviewing (CAPI) to collect data. Only those who provided voluntary consent were interviewed. The published survey report describes the survey design, questionnaire, quality control measures, and survey management information in greater detail [23]. The current study used data from women aged 15–49 years who had had their last pregnancy terminated by induced abortion in the five years preceding the survey (n = 5,835).

### Outcome variable

The outcome variable of this study was the experience of any post-abortion complication. In the survey, women whose last pregnancy during the last five years ended in abortion were asked – ‘Did you have any complication from the abortion?’. Women reporting ‘yes’ to this question were considered to have post-abortion complications.

### Predictor variables

The individual, household, community, and facility-level predictor variables used in the analysis were based on a literature review and information available in the NFHS-5. Individual-level characteristics included were women’s age in years (15–24, 25–34, 35–49), gestational age in weeks ( < = 8, 9–20, >=20), years of schooling (no schooling, < 10 years, >=10 years) and reasons for the abortion (Life risk/medical reasons, sex-selective abortions, unintended pregnancies). The analysis also took into account household characteristics like social group (scheduled caste/scheduled tribe-SC/ST, other backward classes-OBC, Non-SC/ST/OBC), wealth quintile (poorest, poorer, middle, richer, richest), and community-level characteristics such as place of residence (rural, urban), and geographical regions (North, Central, East, North East, West, South). Facility and provider-level features like the method of abortion (medicine, manual vacuum aspiration-MVA, other surgical, other/do not know), place of abortion (public, private, home/other), and the person who performed the abortion (doctor, other health care provider-HCP, self) were also included. Abortions before nine gestational weeks are suggested to be carried out through medicine, while abortions in the advanced gestational weeks require more invasive procedures, and beyond 20 weeks’ abortions are more likely to be performed due to major health complications [24].

### Statistical analysis

For this study, the information related to abortion was extracted from the calendar data available in the individual file of the NFHS-5 data set. Data on women who had their last pregnancy terminated by induced abortion in the five years preceding the survey were retained and analysed. The socioeconomic, demographic, and health profiles of women aged 15–49 who terminated their last pregnancy with abortion were presented using descriptive analysis. Bivariate analyses were performed to assess the individual association between the outcome variable and the predictors. Multivariate logistic regression was conducted to examine the adjusted association of socioeconomic and demographic characteristics with post-abortion complications. The predictor variables used in the regression study were chosen after a collinearity check using the Variance Inflation Factor (VIF) method. Sample weights were used to adjust the non-response. The analyses were performed using Stata, and the findings were presented at a 5% significance level.

## Results

### Profile of abortion-seeking women

Table 1 presents the characteristics of women who ended their last pregnancy with an abortion during the five years preceding the survey. Of the total sample women, 59% were 25–34 years old, 22% were 35–49 years old, and the remaining 19% were 15–24 years old. Nearly three-fourths (73%) of women, while obtaining an abortion, had a gestational age of fewer than eight weeks, 25% had a gestational age of 9–20 weeks, and 2% had a gestational age of more than 20 weeks. About two-thirds (68%) of women took medication, 16% used another surgical method, 12% had manual vacuum aspiration (MVA), and only 5% used other methods/did not know the method of abortion. Over half (53%) of the women underwent an abortion in a private health facility, 27% at home/others, and 20% in the public sector. Doctors performed over half of the abortions (55%), followed by self-aborts (27%) and other healthcare providers (19%). More than two-thirds (71%) of the women who had abortions stated unintended pregnancy as their prime reason, followed by 22% who cited a life risk and 7% who cited sex selection. Of the women, 44% attended school for 10 or more years, 41% for less than 10 years, and the remaining 15% did no schooling. About two-fifths (41%) of the women belonged to OBC, 30% to Non-SC/ST/OBC, and 29% to SC/ST category. Of the women, 23% were from richest, 22% from the richer, 21% from the middle, 20% from the lower, and 15% from the lowest wealth quintile. The majority of these women (62%) lived in rural areas. Of these women, 27% belong to the central region, 26% to the eastern region, 16% to the southern region, 14% to the western region, 11% to the Northern region, and 7% to the North-eastern region.

**Table 1:** Profile of the women aged 15–49 who terminated their last pregnancy with an abortion in the five years prior to the survey, India, 2019-21 (n = 5835)

Characteristics	Women who had an abortion	
	%	n
**Age**		
15–24	18.75	1094
25–34	59.28	3459
35–49	21.97	1282
**Gestational age**		
<=8 weeks	72.72	4243
9–20 weeks	25.04	1461
>=20 weeks	2.24	131
**Method of abortion**		
Medicine	67.74	3953
MVA	11.62	678
Other surgical	16.03	935
Other/Do not know	4.61	269
**Place of abortion**		
Public	20.44	1193
Private	53.03	3094
Home/Other	26.53	1548
**Person conducted abortion**		
Doctor	54.5	3181
Other health care providers	19.0	1108
Self	26.5	1546
**Reasons for abortion**		
Life-risk/medical reasons	21.8	1270
Sex-selective	7.0	410
Unintended pregnancy	71.2	4155
**Years of schooling**		
No schooling	15.04	877
< 10	41.34	2412
>=10	43.62	2546
**Caste**		
SC/ST	29.04	1694
OBC	40.92	2388
Non-SC/ST/OBC	30.04	1753
**Wealth quintile**		
Poorest	14.67	856
Poorer	19.84	1158
Middle	20.91	1220
Richer	21.56	1258
Richest	23.02	1343
**Place of residence**		
Urban	37.91	2212
Rural	62.09	3623
**Region**		
North	11.12	649
Central	26.90	1570
East	25.66	1497
North East	6.66	389
West	13.81	805
South	15.85	925
**Total number of women**	**100**	**5835**

### Abortion complications by background characteristics

The data in Table 2 reveals that 16% of the women had post-abortion complications. More than one-fifth (23%) of the women with a gestational age of 9–20 weeks had abortion complications compared with 18% of those with a gestational age of 20 or more weeks. Again, compared to 15% of women who used abortion medications, 23% of women who had MVA encountered abortion complications. Abortion complications affected 18% of women who had an abortion at a private health facility, 16% in a public health facility, and 14% at home or another location. Similarly, 17% of women who were aborted by a doctor and 17% by other health care providers had abortion complications compared to 14% who self-aborted. One-fifth (20%) of women whose reason for abortion was life risk/medical reasons had abortion complications compared to that 15% whose reason was unintended pregnancy. Complications from abortion affected 20% of those with no schooling, 17% with ten or more years of schooling, and 15% with less than ten years. Nearly one-fifth (18%) of those belonging to Non-SC/ST/OBC had abortion complications compared to 16% of those SC/ST or OBC. Again, 18% of women from the poorer wealth quintile had abortion complications compared to 14% from the richest wealth quintile. Similarly, 18% of women from the northern and western regions had abortion complications compared to 14% from the southern region.

**Table 2:** Percentage of women aged 15–49 whose last pregnancy terminated in an abortion experienced post-abortion complication by background characteristics, India, 2019-21 (n = 5835)

Characteristics	Post-abortion complications (%)	n
**Age**		
15–24	17.9	1094
25–34	15.7	3459
35–49	16.8	1282
**Gestational age**		
<=8 weeks	14.0	4243
9–20 weeks	23.0	1461
>=20 weeks	18.1	131
**Method of abortion**		
Medicine	14.7	3953
MVA	23.3	678
Other surgical	18.2	935
Other/Do not know	16.2	269
**Place of abortion**		
Public	15.7	1193
Private	17.7	3094
Home/Other	14.0	1548
**Person conducted abortion**		
Doctor	17.2	3181
Other health care providers	16.8	1108
Self	14.3	1546
**Reasons for abortion**		
Life-risk/medical reasons	19.9	1270
Sex-selective	19.3	410
Unintended pregnancy	15.0	4155
**Years of schooling**		
No schooling	20.4	877
< 10	14.7	2412
>=10	16.5	2546
**Caste**		
SC/ST	15.7	1694
OBC	15.6	2388
Non-SC/ST/OBC	18.0	1753
**Wealth quintile**		
Poorest	15.7	856
Poorer	17.9	1158
Middle	17.0	1220
Richer	17.5	1258
Richest	13.6	1343
**Place of residence**		
Urban	15.5	2212
Rural	16.8	3623
**Region**		
North	17.9	649
Central	15.8	1570
East	17.1	1497
North East	15.7	389
West	17.9	805
South	13.8	925
**Total number of women**	**16.3**	**5835**

### Determinants of abortion complications

Table 3 presents the crude and adjusted odds ratio for post-abortion complications. The crude odds of abortion complication were higher for women who had an abortion with a gestational age of 9–20 weeks (COR:1.66, CI: 1.41–1.94) or 20 and above weeks (COR: 1.80, CI: 1.16–2.79) than those with a gestational age of < = 8 weeks. After adjusting the effects of the model’s predictors, the multivariate logistic regression revealed that the women who had an abortion with a gestational age of 9–20 weeks (AOR:1.48, CI: 1.24–1.75) were more likely to have complications than those with a gestational age of < = 8 weeks. The chances of complication were again higher among those who terminated their pregnancy through surgical methods (COR: 1.33, CI: 1.10–1.60) than their counterparts who used medicine for terminating the pregnancy. Women having an abortion in public (COR: 1.39, CI:1.13–1.70) or private health facility (COR: 1.33, CI: 1.10–1.59) were more likely to have any complications than their counterparts who reported home as the place of abortion. Self-conducted abortions had lower odds of complication (COR: 0.77, CI: 0.64–0.92) than those conducted by a doctor. However, the statistically significant association of method, person, and place of abortion with abortion complications disappeared when adjusted for socio-economic and demographic characteristics. Women who had an abortion due to life risk/medical reasons had higher odds of abortion complications (COR:1.61, CI:1.36–1.90) than those who had an abortion due to unintended pregnancy. This association remained significant after controlling the effects of socio-economic and demographic characteristics (AOR:1.37, CI:1.13–1.65). The crude odds ratio reveals that the chances of complications were low among the women from central (COR: 0.76, CI: 0.60–0.96), eastern (COR: 0.77, CI: 0.61–0.98), north east (COR: 0.66, CI: 0.52–0.84) and the south region (COR: 0.68, CI: 0.52–0.90) than their counterparts from the north. However, when adjusted for the socio-economic and demographic characteristics, only the women from the North-Eastern (AOR:0.67, CI:0.51–0.88) and the Southern (AOR:0.60, CI:0.44, 0.81) regions were less likely to have abortion complications than those from the Northern region.

**Table 3:** Crude and adjusted odds ratio for post-abortion complications among women aged 15–49, India, 2019-21

Predictor variables	COR (95% CI)	AOR (95% CI)
Characteristics		
**Age**		
15–24**®**		
25–34	0.85 (0.70, 1.03)	0.85 (0.70, 1.04)
35–49	0.93 (0.74, 1.16)	0.91 (0.72, 1.15)
**Gestational age**		
<=8 weeks**®**		
9–20 weeks	1.66*** (1.41, 1.94)	1.48*** (1.24, 1.75)
>=20 weeks	1.80** (1.16, 2.79)	1.48 (0.93, 2.34)
**Method of abortion**		
Medicine**®**		
MVA	1.20 (0.96, 1.50)	1.04 (0.82, 1.33)
Other surgical	1.33** (1.10, 1.60)	1.11 (0.90, 1.38)
Other/Don’t know	0.98 (0.65, 1.46)	0.83 (0.55, 1.26)
**Place of abortion**		
Home/Other ®		
Public	1.39** (1.13, 1.70)	1.18 (0.88, 1.60)
Private	1.33** (1.10, 1.59)	1.18 (0.89, 1.55)
**Person conducted abortion**		
Doctor**®**		
Other health care providers	1.07 (0.88, 1.29)	1.05 (0.85, 1.30)
Self	0.77** (0.64, 0.92)	1.04 (0.77, 1.39)
**Reasons for abortion**		
Unintended pregnancy®		
Life-risk/medical reasons	1.61*** (1.36, 1.90)	1.37*** (1.13, 1.65)
Sex-selective	1.24 (0.93, 1.64)	1.15 (0.86, 1.53)
**Years of schooling**		
No schooling**®**		
< 10	0.82 (0.66, 1.01)	0.86 (0.69, 1.08)
>=10	0.84 (0.68, 1.04)	0.90 (0.70, 1.15)
**Caste**		
Non-SC/ST/OBC®		
SC/ST	0.93 (0.77, 1.11)	0.95 (0.78, 1.15)
OBC	0.82* (0.68, 0.99)	0.85 (0.70, 1.03)
**Wealth quintile**		
Poorest**®**		
Poorer	1.07 (0.85, 1.36)	1.10 (0.86, 1.41)
Middle	1.17 (0.92, 1.49)	1.17 (0.90, 1.52)
Richer	1.00 (0.78, 1.28)	0.97 (0.72, 1.29)
Richest	0.98 (0.76, 1.26)	0.89 (0.64, 1.24)
**Place of residence**		
Urban**®**		
Rural	1.12 (0.95, 1.33)	1.08 (0.89, 1.30)
**Region**		
North**®**		
Central	0.76* (0.60, 0.96)	0.82 (0.64, 1.05)
East	0.77* (0.61, 0.98)	0.81 (0.63, 1.06)
North East	0.66** (0.52, 0.84)	0.67** (0.51, 0.88)
West	0.99 (0.73, 1.34)	1.00 (0.73, 1.37)
South	0.68** (0.52, 0.90)	0.60*** (0.44, 0.81)

Note: ® Reference category; * p < .05; ** p < .01; *** p < .001

## Discussion

The study found that many women had post-abortion complications, and the prevalence varied considerably by socioeconomic and demographic characteristics. Gestational age, reasons for abortion, and region are significantly associated with post-abortion complications. We found that women who had an abortion with a gestational age of 9–20 weeks had a higher risk of abortion complications, similar to previous studies indicating that complication rates invariably increase with increased gestational age [25–27]. Another study revealed that women who had undergone vacuum aspiration abortions were shown to have a measurable increase in post- abortion hemorrhage with increasing gestational age, and this rise became noticeable after 11 weeks of gestation [28]. A past study also found that the odds ratio for complications was more likely between 9 and 12 weeks of gestation compared to the complications that occurred at < 9 weeks and increased dramatically with a further increase in gestational age [29]. Most likely, late abortions involve sophisticated, specialized procedures; nevertheless, other elements, like the medical staff’s expertise, the availability of backup facilities, and more modern technology, may also play a role. However, an earlier study revealed that post-abortion hemorrhage or fever risk does not significantly differ at gestational age < 10 weeks and ≥ 10 weeks [30]. The difference in study settings and the outcome measure are the reasons for this disagreement. Our study includes all women who had an induced abortion, irrespective of the method of abortion during the five years prior to the survey, while the referred study is based on surgical abortions performed > 30 days prior to the survey.

This study found that women in the north-eastern and southern regions are less likely to have abortion complications. The regional disparity may be caused by differences in the access and quality of abortion care services [8]. Female literacy is known to be better among the southern and north-eastern regions of the country [31], which might help utilize timely and appropriate care from competent providers/facilities, subsequently reducing abortion complications. Additionally, the southern region is proven to have a better health infrastructure [32], thus reducing possible complications from abortions. A past study found that a higher proportion of the facilities providing any abortion care services in the state of Assam in the northeastern region and Tamil Nadu in the Southern region provide both abortion and post-abortion care services than those states of Bihar, Uttar Pradesh, Gujarat and Madhya Pradesh [33].

Our study revealed that women who had abortions for life-risk or medical reasons are more likely to experience complications. This finding may be related to the fact that more women are given foetal anomaly diagnoses, usually in the second trimester of pregnancy, and that parents may end up considering a medically based termination when a mother’s life is significantly in danger if she chooses to carry on with the pregnancy due to a rare pregnancy or other health issues [34]. A study by Mentula et al., 2011 [27] found that the second-trimester medical termination of pregnancy had a higher risk of surgical evacuation and infection than the first-trimester medical termination.

The strength of this study is the large sample size from a recent nationally representative survey with a reliable sampling design. The study provides information on the prevalence and predictors of post-abortion complications at the national level. Understanding the predictors of abortion complications helps us better understand the relationship between those complications and women’s socioeconomic level and demographic characteristics. The result is recent and relevant for interventions in policies and programs that emphasize offering quality abortion care. However, the survey’s cross-sectional design restricts the causal association between the predictors and outcome variable. Abortion complication is self-reported, and the estimate is based on the answer to a single question without capturing the specific complication women underwent; thus, there is a possibility of over/underestimation. Additionally, because abortion is still a sensitive subject, social desirability response bias cannot be ruled out. The data collection in the second phase of the NFHS-5 coincided with COVID-19-induced disruption in the provision of essential reproductive healthcare, including abortion services. This may have also impacted the results of the study.

## Conclusion

A sizable number of Indian women experience post-abortion complications, with the primary causes being increased gestational age and abortions performed due to life-threatening or medical conditions. Additionally, there are regional variations in the post-abortion complications. Efforts to educate women about early abortion decision-making and improve abortion care will reduce post-abortion complications. Moreover, abortions, if required for life-saving, should be carried out under the supervision of a qualified medical professional, with the support of contemporary technology. Improved abortion care will ensure women’s reproductive rights and help attain Sustainable Development Goal 3, “Good Health and Well-being.“

## Data Availability

The datasets generated and/or analyzed during the current study are available in the Demographic and Health Surveys Repository [https://www.dhsprogram.com/data/new-user-registration.cfm].

## List of abbreviations

MTP: Medical Termination of Pregnancy
NFHS: National Family Health Survey
PPS: Probability Proportional to Size
CAPI: Computer-Assisted Personal Interviewing
VIF: Variance Inflation Factor
SC/ST: Scheduled Caste/Scheduled Tribe
OBC: Other Backward Classes
MVA: Manual Vacuum Aspiration
HCP: Health Care Provider
COR: Crude Odds Ratio
AOR: Adjusted Odds Ratio
